# Genes to Diseases (G2D) Computational Method to Identify Asthma Candidate Genes

**DOI:** 10.1371/journal.pone.0002907

**Published:** 2008-08-06

**Authors:** Karine Tremblay, Mathieu Lemire, Camille Potvin, Alexandre Tremblay, Gary M. Hunninghake, Benjamin A. Raby, Thomas J. Hudson, Carolina Perez-Iratxeta, Miguel A. Andrade-Navarro, Catherine Laprise

**Affiliations:** 1 Department of Medicine, Laval University, Québec, Quebec, Canada; 2 University of Montreal Community Genomic Centre, Chicoutimi Hospital, Saguenay, Quebec, Canada; 3 Ontario Institute for Cancer Research, Toronto, Ontario, Canada; 4 Channing Laboratory, Brigham and Women's Hospital, Harvard Medical School, Boston, Massachusetts, United States of America; 5 McGill University and Genome Quebec Innovation Centre, Montreal, Quebec, Canada; 6 Molecular Medicine, Ottawa Health Research Institute, Ottawa, Ontario, Canada; 7 Faculty of Medicine, University of Ottawa, Ottawa, Ontario, Canada; 8 Max Delbrück Center for Molecular Medicine, Berlin, Germany; 9 Département des Sciences fondamentales, Université du Québec à Chicoutimi, Saguenay, Quebec, Canada; Leiden University Medical Center, Netherlands

## Abstract

Asthma is a complex trait for which different strategies have been used to identify its environmental and genetic predisposing factors. Here, we describe a novel methodological approach to select candidate genes for asthma genetic association studies. In this regard, the Genes to Diseases (G2D) computational tool has been used in combination with a genome-wide scan performed in a sub-sample of the Saguenay−Lac-St-Jean (SLSJ) asthmatic familial collection (n = 609) to identify candidate genes located in two suggestive loci shown to be linked with asthma (6q26) and atopy (10q26.3), and presenting differential parent-of-origin effects. This approach combined gene selection based on the G2D data mining analysis of the bibliographic and protein public databases, or according to the genes already known to be associated with the same or a similar phenotype. Ten genes (*LPA*, *NOX3*, *SNX9*, *VIL2*, *VIP*, *ADAM8*, *DOCK1*, *FANK1*, *GPR123* and *PTPRE*) were selected for a subsequent association study performed in a large SLSJ sample (n = 1167) of individuals tested for asthma and atopy related phenotypes. Single nucleotide polymorphisms (n = 91) within the candidate genes were genotyped and analysed using a family-based association test. The results suggest a protective association to allergic asthma for *PTPRE* rs7081735 in the SLSJ sample (p = 0.000463; corrected p = 0.0478). This association has not been replicated in the Childhood Asthma Management Program (CAMP) cohort. Sequencing of the regions around rs7081735 revealed additional polymorphisms, but additional genotyping did not yield new associations. These results demonstrate that the G2D tool can be useful in the selection of candidate genes located in chromosomal regions linked to a complex trait.

## Introduction

Asthma involves genetic and environmental factors in its development, chronicity and severity [1], [2]. Although some of its underlying mechanisms have been elucidated in recent years, more work is needed to gain a clearer understanding of genetic determinants. The mapping of asthma has been one of the most important areas of human genetics in the last two decades. According to an overview by Blumenthal (2005), twelve complete and two incomplete genome scans for asthma have been published, identifying a total of twenty chromosomal linked regions to asthma [3]. Discrepancies often appeared between linkage studies [3]–[5], leading to the use of standard phenotype definitions and founder populations as a way to decrease phenotypic and genetic heterogeneity [6]–[8].

To date, common strategies have been employed to identify genes involved in asthma predisposition. Linkage studies followed by positional cloning identified six genes while association studies identified over a hundred genes, the majority of these having quite small effects on asthma susceptibility (see [9], [10] for a review). Here, we describe a novel methodological approach that combines classical genetic approaches with a computational data-mining tool. In this regard, a genome-wide scan for asthma and atopy in families originating from the Saguenay–Lac-St-Jean (SLSJ) founder population (Northeastern Quebec, Canada) [11]–[16] has been combined with the Genes to Diseases (G2D) new computational tool in the prioritization of asthma candidate genes [17], [18]. G2D performs the selection of the candidates in the chromosomal regions genetically linked to a disease by highlighting genes whose functions are related to the phenotype of the disease according to a data mining analysis of the bibliographic and protein public databases, or according to the genes already known to be associated with the same or a similar phenotype.

Single nucleotide polymorphisms (SNPs) within the candidate genes prioritized by the G2D tool have been subsequently used to conduct an association study with asthma, atopy and allergic asthma phenotypes in the SLSJ asthma familial collection. The chromosomal regions around associated SNPs have then been sequenced in order to find causal mutations, and a replication of the positive association findings has been assessed in the Childhood Asthma Management Program (CAMP) independent cohort. The goal was to apply the G2D tool in the search of genetic determinants associated with a complex trait, using asthma as a model. This approach led to the hypothesis-driven identification of ten genes that may not have been selected otherwise (*LPA*, *NOX3*, *SNX9*, *VIL2*, *VIP*, *ADAM8*, *DOCK1*, *FANK1*, *GPR123* and *PTPRE*). Of these, one positive genetic association, resisting to corrections for multiple testing, has been found between the protein tyrosine phosphatase receptor type E gene (*PTPRE*) and allergic asthma in the SLSJ sample.

## Methods

### Subjects

Clinical evaluation and phenotyping criteria of the Saguenay-Lac-St-Jean (SLSJ) subjects have been described in recent reports [19]–[21] and summarized in Table 1. This familial sample is predominantly composed of probands that reported an onset age of asthma below 12 years old (81.6% of the probands). The mean age of onset for the probands is 7 years and the mean age of onset for the asthmatic family members is 22 years. The entire sample has been used for the association study while for the genome scan, the first 79 recruited families, that were available at the time when the genome scan was performed have been used (n = 609 individuals–see supplementary Table S1 for subjects characteristics and studied phenotypes). The Chicoutimi Hospital local ethics committee approved the study and all subjects provided informed consent.

**Table 1:** Clinical and phenotypic characteristics of the Saguenay–Lac-St-Jean association study sample subjects.

	Probands (n = 226)	Family members (n = 941)
Male: Female ratio	1: 1.2	1: 1.1
Mean age in years (range)	18 (3–46)	44 (2–96)
Smoking status (n (%))		
Never	185 (83.3)	424 (45.8)
Ex-smoker	12 (5.4)	307 (33.2)
Smoker	25 (11.3)	195 (21.0)
FEV_1_ % of predicted value (SD) *	92.3 (16.3)	94.4 (20.5)
PC_20_ in mg/ml (SD) †	2.68 (3.59)	10.87 (5.23)
Serum IgE in µg/l (SD) ‡	233.2 (4.6)	108.0 (4.2)
Number of persons with subphenotypes (%)		
Asthma §	226/226 (100)	353/935 (37.8)
Atopy ∏	185/224 (82.6)	468/920 (50.9)
Allergic asthma (Asthma+Atopy)	185/224 (82.6)	239/344 (69.5)

* FEV_1_ = Geometric mean of the force expiratory volume in one second evaluated for 208 probands and for 683 family members.

† PC_20_ = Concentration of methacholine inducing a 20% fall in FEV1. Geometric mean and SD were obtained from the log transformed PC20 values. Evaluated for 186 probands and for 614 family members.

‡ IgE = Immunoglobulin E serum concentration. Geometric mean and SD were obtained from the log transformed IgE values. Evaluated for 211 probands and 704 family members.

§ Present asthma or past documented clinical history of asthma. The reported mean age of onset is 7 years among the asthmatic probands and 22 years among the asthmatic family members.

∏ Defined as at least one positive response on skin prick testing (wheal diameter >3 mm at 10 minutes).

### Genome Scan

DNA was extracted for all SLSJ participants from whole blood by using the QIAGEN genomic purification procedure (QIAGEN Inc., Valencia, CA). Genotyping was completed on 367 autosomal and 21 X-chromosome microsatellite markers evenly spaced throughout the genome (average spacing of 9.2 cM). The marker set is a modification of the Cooperative Human Linkage Centre Screening Set (http://gai.nci.nih.gov/CHLC/, version 6.0), showing an average heterozygosity of 0.72 in our data set. Each primer was amplified separately and then pooled into panels of eight markers and products were interrogated using ABI 3700 sequencers (Applied Biosystems, Foster City, CA) with a size standard ladder. Duplicate of two CEPH control DNAs and one water were included in each genotyping plate. In our data set of 195939 autosomal genotypes, 91.4% of the alleles were called and the proportion of observed Mendelian error was 1.2%. Linkage of chromosomal regions with putative genetic risk factors for a given trait was assessed by evaluating the extent of excess sharing of alleles identical by descent in affected relatives within families. Test statistics are reported on the LOD scale. Briefly, a multipoint, one-parameter likelihood ratio test that is robust against incompleteness of marker data (when the descent of alleles in a pedigree is not fully known) was used [22], [23]. We moreover evaluated the specific contribution of mothers and fathers to the test of linkage, to look for parent-of-origin effects. The above tests of linkage, linkage through mothers and linkage through fathers are described in details in [24].

### Genes to Diseases (G2D)

The G2D tool has been applied in the two best genome scan susceptibility regions: 6q26 between markers D6S476 and D6S305, and 10q26.3 between markers D10S1223 and D10S1248. We used two of the approaches considered in G2D [18] to pre-select gene lists in these regions. The first approach uses a description of the phenotype to point to genes in a region. This method works with automatically derived relationships between the disease symptoms (as MeSH C terms) and gene features (as Gene Ontology or GO terms [25]) that are obtained from the literature and Entrez gene database (http://www.ncbi.nlm.nih.gov/). We call this procedure the “PHENOTYPE” method. The second approach consists on automatically finding genes in a region that are similar to other genes previously associated to asthma. To do this, G2D measures the semantic distance between the annotations of the “known genes” and the annotations of genes in the problem region assigned by homology searches. We call this procedure the “KNOWN GENES” method. For details about the algorithm see the G2D web site at http://www.ogic.ca/projects/g2d_2/ and [17], [18].

### Association Study

#### SNP selection

The HapMap database (http://www.hapmap.org/) has been used to identify SNPs assumed to be polymorphic in the SLSJ population. TagSNPs were then selected with the tagger program implemented in the Haploview software (version 3.32)[26] using an r^2^ cutoff of 0.8 and a minor allele frequency (MAF) over 0.10 to cover each whole gene. SNPs were also prioritized on their localization (coding or untranslated regions-see supplementary Table S2). All SNPs are referred using their reference sequence number (rs#).

#### SNP genotyping

Eighty SNPs have been genotyped by the Sequenom® matrix-assisted laser desorption/ionization time-of-flight mass array spectrometer (Sequenom Inc., San Diego, CA) (Table S2). Sequenom primers were designed using the Sequenom SNP Assay Design software version 3.0 for iPLEX reactions. A total of 74 assays were designed for a single multiplex reaction. The assay group file containing the PCR primers and the iPLEX extension probes can be supplied on request to the corresponding author. The protocol and reaction conditions are in accordance with the manufacturer [27]. The genotypes were viewed and analyzed using the MassARRAY Typer software version 3.4 (Sequenom Inc., San Diego, CA). The ten remaining SNPs (Table S2) have been genotyped by the TaqMan® SNP Genotyping Assays (Applied Biosystems, Foster City, CA) using the Rotor-Gene™ real-time PCR (Corbett Research Ltd, Sydney, Australia). Protocol and method were supplied by the manufacturer and PCR conditions were optimized to get a good cluster separation between different genotypes (see supplementary Table S3 for PCR conditions). Genotypes were attributed by the Rotor Gene software using the scatter graph analysis option.

#### Statistical analysis

Family-based association testing has been performed with the FBAT software (version 1.7) using an empirical estimate of the variance [28]–[30] to correctly account for linkage. A Sidak correction for multiple testing has been applied on the p-values accounting for the effective number of independent phenotypes and SNPs, according to the definition of Li and Ji [31], as implemented in the SNPSpD program [32] (see the online supporting Text S1 file for supplementary details). Parent-specific transmission disequilibrium tests were performed using sib_tdt from the ASPEX package (http://aspex.sourceforge.net/). Mendelian errors have been assessed by FBAT and Hardy-Weinberg equilibrium has been assessed with Haploview software (version 3.32)[26].

### *PTPRE* Sequencing

Forty unrelated SLSJ subjects (validated with the BALSAC database [33]) presenting full-fit allergic asthmatic criteria and that have contributed to the *PTPRE* association were selected. *PTPRE* sequence information was obtained from Ensembl database (http://www.ensembl.org/index.html, release 46). The sequencing was divided in six regions that spanned 2.7 kb, starting from the exon 2 to the exon 4. Oligonucleotides and PCR conditions are listed in Table S4. Amplification products were purified with multiscreen PCR plates (Millipore Corporation, Billerica, MA), sequenced with BigDye terminator v3.1 chemistry following instructions of the manufacturer and analyzed on a 3100 Genetic analyzer (Applied Biosystems, Foster City, CA). Sequence analysis was performed with Codoncode Aligner software (CodonCode Corporation, http://www.codoncode.com/). The identified SNPs presenting MAF over 0.05 have been genotyped in the SLSJ sample using a Sequenom panel and analyzed with FBAT, as described above. Newly described SNPs have been submitted to NCBI (http://www.ncbi.nlm.nih.gov/) SNP database.

### Replication Study

The *PTPRE* rs7081735 association has been assessed in the Childhood Asthma Management Program (CAMP) study [34], [35]. This analysis includes the 497 non-Hispanic white children and their parents for whom adequate DNA was available. The genotyping of the *PTPRE* rs7081735 was performed using a TaqMan® SNP Genotyping Assays (Applied Biosystems, Foster City, CA) (see Table S3 for PCR conditions). Plates were scanned using the 7900HT Fast Real-Time PCR System (Applied Biosystems, Foster City, CA) and genotypes were assigned by the SDS 2.2 software using the scatter graph analysis option. The Institutional Review Board of the Brigham and Women's Hospital (BWH), as well as those of the other CAMP study centers, approved this study. Informed assent and consent were obtained from the study participants and their parents to collect DNA for genetic studies.

## Results

### Genome Scan

**Figure 1:**
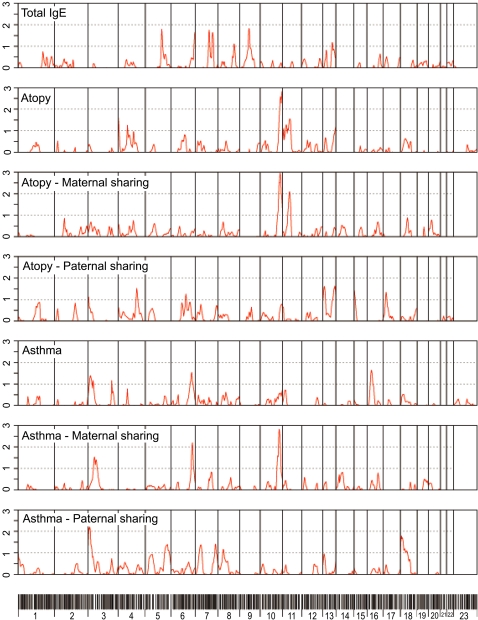
Genome scan results summary. Results from the tests of linkage with atopy (top three panels) and asthma (bottom three panels) reported on the LOD scale. For each phenotype, the top panel shows the results of the test of linkage (excess allele sharing), the middle panel shows results from the tests of linkage through mothers (excess allele sharing transmitted from mothers) and the bottom panel shows results from the tests of linkage through fathers (excess allele sharing transmitted from fathers).

The genome-wide linkage scan analysis revealed at least two regions showing suggestive evidence for linkage, as well as differential maternal and paternal contribution (Figure 1). The two regions are 6q26 for asthma (LOD = 1.54, p = 0.0038) and 10q26.3 for atopy (LOD = 2.82, p = 0.00016). In these two regions, affected sibs tend to share more alleles inherited from the mothers than from the fathers. The linkage tests through the mothers reach a LOD of 2.19 (p = 0.00074) in 6q26 for asthma and a LOD of 2.96 (p = 0.00011) in 10q26.3 for atopy. The respective LODs obtained through the fathers at the same loci are only 0.01 (p = 0.40) and 0.58 (p = 0.05). Moreover, even though the 10q region does not show a great strength of linkage with asthma (LOD = 0.57, p = 0.051), the asthmatic sibs tend to share these alleles when received from their mothers (LOD = 2.82, p = 0.00016). Each of the two chromosomal regions show LOD values (either for tests of linkage, or parent-specific LODs, or both) that are, in order of magnitude, consistent with what has been defined as «suggestive» for linkage by Lander and Kruglyak [36], which are expected to occur once per whole genome scan on average. Because of their great hypothesis generating potential, the 6q26 and 10q26 regions as well as asthma and atopy phenotypes were selected for the following G2D and association studies.

### G2D

We applied the two algorithms PHENOTYPE and KNOWN GENES to the 6q26 and 10q26.3 regions. For the PHENOTYPE method, we used the OMIM [37] record 600807 as input, particularly the MeSH C terms from the MEDLINE references in OMIM entry 600807 which deals with susceptibility to asthma and asthma related traits. The most prevailing MeSH C terms are “Asthma” and “Bronchial Hyperreactivity”, but the list also includes “Hypersensitivity”, “Respiratory Hypersensitivity” and “Eosinophilia”. We thus considered that the terms that refer to asthma were “Asthma” and “Bronchial Hyperreactivity” and that the terms that refer to atopy were “Hypersensitivity”, “Respiratory Hypersensitivity” and “Eosinophilia”. The highest scoring GO terms, associated to these MeSH C terms, describe a variety of molecular functions and processes that include leukotriene and interleukin signaling, glutathione metabolism, etc (see header of supplementary Table S5). We applied this method to the 6q26 region between D6S476 and D6S305, the two markers directly flanking the linkage peak seen in the region, which corresponds to the 10.48 MB band between positions 151,685,574 and 162,165,587 of chromosome 6. After discarding candidates that did not overlap with any known or hypothetical Entrez Gene sequence, 16 genes remained (see supplementary Table S5-A). A similar analysis was carried between D10S1223 and D10S1248, the two markers directly flanking the linkage peak in 10q26.3, between positions 129,150,822 and 130,982,363 in chromosome 10. In that case, 12 candidates were obtained (see supplementary Table S5-B). For the KNOWN GENES method, we compiled a list of genes reported to be associated with asthma and atopy from the literature [38] and from the Genetic Association Database GAD [39] (supplementary Table S6). We then extracted the GO annotation of those genes in Entrez Gene [37]. We derived a scoring system for the candidates according to the minimal semantic distance between their GO annotation and the ones from the compiled known-gene list. For example, genes annotated with GO terms such as “dipeptidyl-peptidase IV activity” or “chemokine receptor binding” would score high as candidates. The method takes into account the hierarchical structure of GO as well as the specificity of GO terms. In that sense, similarity with more infrequent terms receive higher scores. For the complete list of GO terms see the header of supplementary Table S7. We applied the KNOWN GENES method to both regions 6q26 and 10q26.3, obtaining 15 and 10 genes, respectively, after filtering out those candidates that did not overlap with either known or hypothetical genes (see supplementary Table S7).

#### Use of a complementary method based on genomic sequence

We applied the G2D complementary Disease Gene Prediction (DGP) tool [40] to both genetically linked regions in order to predict the involvement of genes in inherited disease by their sequence features. This method analyses the probability of a gene to be associated with any disease phenotype. Genes with no associated phenotype and a probability greater than 0.7 were retained. With this criterion the DGP method identified one candidate in chromosome 10 (MMP21) and three candidates in the 6q26 region: *TFB1M* [MIM:607033], *RGS17* [MIM:607191] and *VIP* [MIM:192320].

#### Final list of candidate genes

Genes that received the higher scores in the pre-selected lists, and those that were pointed by the two different analyses were preferred, allowing the construction of a list of 17 candidates (displayed in the Table 2). We then applied a candidate gene approach to select a final list of ten genes with the best biological potential related to asthma pathophysiology for genotyping (five in each chromosomal region): *LPA*, *NOX3*, *SNX9*, *VIL2*, *VIP*, *ADAM8*, *DOCK1*, *FANK1*, *GPR123* and *PTPRE* (marked in bold in Table 2).

**Table 2:** Genes identified by G2D data mining analysis and those selected for the association study based on their number of appearance in G2D analyses and on their biological function.

Gene *	GeneID/[MIM]	Analysis †	Gene definition ‡	Gene function ‡
*ARID1B* 6q26	57492/[MIM:NA]	No	AT rich interactive domain 1B (SWI1-like)	Involved in transcriptional activation and repression of select genes by chromatin remodeling (alteration of DNA-nucleosome topology). Binds DNA non-specifically.
*ESR1* 6q26	2099/[MIM:133430]	Yes	Estrogen receptor 1	Nuclear hormone receptor. The steroid hormones and their receptors are involved in the regulation of eukaryotic gene expression and affect cellular proliferation and differentiation in target tissues.
*IGF2R* 6q26	3482/[MIM:147280]	Yes	Insulin-like growth factor 2 receptor	Transmembrane protein with a short cytoplasmic tail containing an internalization signal. This receptor binds insulin-like growth factor IGF2.
***LPA*** 6q26	4018/[MIM:152200]	No	Lipoprotein, Lp(a)	Apo(a) is the main constituent of lipoprotein(a). It has a serine proteinase activity and is able of autoproteolysis. Inhibits tissue-type plasminogen activator 1.
***NOX3*** 6q26	50508 [MIM:607105]	Yes	NADPH oxidase 3	NADPH oxidases, such as NOX3, are plasma membrane-associated enzymes found in many cell types. They catalyze the production of superoxide by a 1-electron reduction of oxygen, using NADPH as the electron donor.
*SLC22A2* (OCT2) 6q26	6582/[MIM:602608]	No	Solute carrier family 22 (organic cation transporter), member 2	Polyspecific transporter of organic cations, mainly expressed in kidney (luminal membrane of distal tube).
***SNX9*** 6q26	51429/[MIM:605952]	No	Sorting nexin 9	This gene encodes a member of the sorting nexin family. Members of this family contain a phox (PX) domain, which is a phosphoinositide binding domain, and are involved in several stages of intracellular trafficking.
*TFB1M* 6q26	51106/[MIM:607033]	No	Transcription factor B1, mitochondrial	The transcription of genes from mitochondrial DNA requires a mitochondrial RNA polymerase and a DNA-binding transcription factor. Transcription factor B1 (TFB1M) is a part of this transcription complex and is implied in the rRNA processing.
***VIL2*** 6q26	7430/[MIM:123900]	No	Villin 2 (ezrin)	The cytoplasmic peripheral membrane protein that functions as a protein-tyrosine kinase substrate in microvilli. Serves as an intermediate between the plasma membrane and the actin cytoskeleton. Plays a key role in cell surface structure adhesion, migration, and organization.
***VIP*** 6q26	7432/[MIM:192320]	No	Vasoactive intestinal peptid	Secreted protein belongs to the glucagon family. It stimulates myocardial contractility, causes vasodilatation, increases glycogenolysis, lowers arterial blood pressure and relaxes the smooth muscle of trachea, stomach and gall bladder.
***ADAM8*** 10q26.3	101/[MIM:602267]	No	ADAM metallopeptidase domain 8	Membrane-anchored proteins structurally related to snake venom disintegrins expressed in granulocyte, monocyte, and macrophage. Implicated in a variety of biological processes involving cell-cell and cell-matrix interactions. May be involved in cell adhesion during neurodegeneration.
*CPXM2* 10q26.3	119587/[MIM:NA]	Yes	Carboxypeptidase X, member 2	Implicated in cell adhesion and proteolysis.
***DOCK1*** 10q26.3	1793/[MIM:601403]	Yes	Dedicator of cytokinesis 1	Role in signaling from focal adhesions. This gene product binds to the SH3 domain of CRK protein. It may regulate cell surface extension and may have a role in the cell surface extension of an engulfing cell around a dying cell during apoptosis.
***FANK1*** 10q26.3	92565/[MIM:611640]	No	Fibronectin type III and ankyrin repeat domains 1	NA
***GPR123*** 10q26.3	84435/[MIM:NA]	No	G protein-coupled receptor 123	Orphan receptor : multi-pass membrane protein.
*MKI67* 10q26.3	4288/[MIM:176741]	Yes	Antigen identified by monoclonal antibody Ki-67	Predominantly localized in the G1 phase in the perinucleolar region and in the nuclear matrix. In mitosis, present on all chromosomes. Thought to be required for maintaining cell proliferation. Regulation of progression through cell cycle.
***PTPRE*** 10q26.3	5791/[MIM:600926]	Yes	Protein tyrosine phosphatase, receptor type E	PTPs are known to be signaling molecules that regulate a variety of cellular processes including cell growth, differentiation, mitotic cycle, and oncogenic transformation.

Genes marked in bold indicate that they have been selected for the association study. NA = Not available.

* Gene symbol, chromosomal location and GeneID number (Entrez Gene identifier in the NCBI database (http://www.ncbi.nlm.nih.gov/)).

† Appears in at least two G2D analyses.

‡ From NCBI (http://www.ncbi.nlm.nih.gov/) and GeneCards (http://www.genecards.org/) databases.

### Association Study

A final panel of 91 SNPs (75 tagSNPs and 16 non-tagSNPs) was selected among the ten candidate genes (supplementary Table S2). Of these, four were non-polymorphic, six failed Sequenom genotyping assays, none presented deviation from Hardy-Weinberg equilibrium (p-values >0.001) and one presented more than two Mendelian errors (Table S2). For the remaining 80 SNPs, genotypes of individuals with Mendelian errors were considered as missing data in the FBAT analyses. Genotyping presented a mean success rate of 99.0% for the Sequenom assays and a mean success rate of 98.3% for the TaqMan assays. Accounting for the residual correlation between the tagSNPs, it is estimated that the 80 partially correlated SNPs correspond to an effective number of 59 independent ones [31]. As for the effective number of phenotypes, simulation indicates that the three studied phenotypes (asthma, atopy and allergic asthma) correspond to 1.75 effective independent ones (see supplementary Text S1 file). Accordingly, the estimated total effective number of independent tests is 103.25 (59 effective independent SNPs×1.75 effective independent phenotypes). Thus, applying Sidak correction, the p-value threshold of significance is estimated to be 0.000483.

The FBAT single marker analyses were performed under an additive genetic model for each SNP and the three studied phenotypes. For the sake of brevity, only results showing a p-value under 0.05 before correction for multiple testing for one or more phenotypes are presented in Table 3. For the asthma phenotype, minor alleles LPA_rs12175867C, GPR123_rs11101913T, GPR123_rs11101932T and GPR123_rs12257731A were overtransmitted to the asthmatic probands, suggesting a susceptibility effect of these alleles for asthma (0.0085<p<0.047). Inversely, minor alleles DOCK1_rs1051039G, PTPRE_rs4369314A and PTPRE_rs7081735G were undertransmitted to the asthmatic probands, suggesting a protective effect of these alleles for asthma (0.010<p<0.049). For the atopy phenotype, only ADAM8_rs11101672G and PTPRE_rs7081735G minor alleles have been undertransmitted to the atopic probands, suggesting a protective effect (p = 0.039 and 0.037, respectively). Finally, for the allergic asthma phenotype, minor alleles LPA_rs12175867C, GPR123_rs11101913T, GPR123_rs11101932T and GPR123_rs12257731A were overtransmitted to the allergic asthmatic probands, suggesting a susceptibility effect of these alleles for allergic asthma (0.035<p<0.041). Inversely, minor alleles ADAM8_rs11101672G, GPR123_rs11101916A, GPR123_rs761777G, PTPRE_rs11016002A, PTPRE_rs4002572C and PTPRE_rs7081735G were undertransmitted to the allergic asthmatic probands, suggesting a protective effect of these alleles for allergic asthma (0.000463<p<0.037). None of the SNPs reported above showed a significantly greater extent of transmission distortion from mothers than from fathers, thus not providing insights to the observed parental distortions seen in the linkage results (not shown).

**Table 3:** Significant Family-Based Association Test (FBAT) results between the ten G2D candidate genes studied SNPs and asthma, atopy and allergic asthma phenotypes under an additive genetic model.

Gene	SNP	Base change	MAF	Asthma			Atopy			Allergic Asthma		
				N	Z	P	N	Z	P	N	Z	P
*LPA*	rs12175867	T>C	0.16	81	1.98	0.0474	68	1.94	0.0530	71	2.04	0.0412
*ADAM8*	rs11101672	C>G	0.23	95	−1.76	0.0784	79	−2.06	0.0390	82	−2.12	0.0340
*DOCK1*	rs1051039	C>G	0.41	117	−1.97	0.0486	101	−0.72	0.4739	103	−0.48	0.6336
*GPR123*	rs11101913	C>T	0.29	112	2.63	0.0085	94	1.81	0.0707	97	2.04	0.0412
	rs11101916	G>A	0.18	95	−1.79	0.0731	73	−1.31	0.1916	80	−2.16	0.0308
	rs11101932	C>T	0.15	80	2.26	0.0240	60	1.74	0.0816	66	2.26	0.0397
	rs761777	A>G	0.29	113	−1.30	0.1948	92	−1.00	0.3152	99	−2.31	0.0209
	rs12257731	G>A	0.12	77	2.15	0.0314	60	1.65	0.0995	65	2.11	0.0353
*PTPRE*	rs11016002	A>T	0.35	113	−1.04	0.2983	89	−1.33	0.1836	93	−2.47	0.0136
	rs4369314	G>A	0.21	96	−2.57	0.0101	81	−1.65	0.0999	83	−1.67	0.0958
	rs4002572	C>T	0.45	113	−1.61	0.1067	86	−1.03	0.3047	93	−2.09	0.0367
	rs7081735	A>G	0.32	111	−2.23	0.0256	88	−2.09	0.0371	91	−3.50	**0.000463**

Abbreviations used: SNP = Single nucleotide polymorphism, MAF = Minor allele frequency, N = Number of families contributing to the statistic, Z = Z score, P = p-value (significance threshold of 0.000483; p-values under are marked in bold).

### *PTPRE* Sequencing

**Figure 2:**
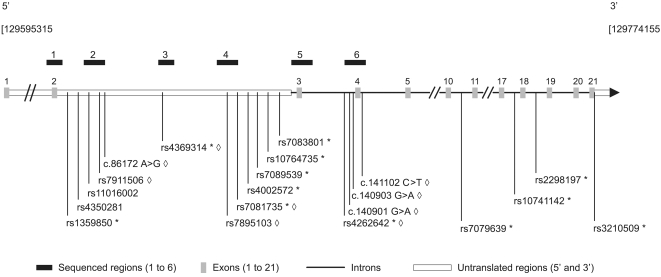
*PTPRE* gene sequenced regions and identified SNPs scaled location. The black thick boxes above the gene define its sequenced parts, which are identified by the same numbers used in the Table 4, in which exact chromosomal positions are available. Studied SNPs are represented below the gene. The TagSNPs correspond to an asterisk (*) and the SNPs identified by sequencing correspond to a diamond (◊). All *PTPRE* numbers for the discovered SNPs are based on the mRNA sequence NM_006504 (variant 1, receptor form) and from the NCBI (http://www.ncbi.nlm.nih.gov/) SNP database (build 127). Image source: HapMap (www.hapmap.org/) October 2007 (Genome Browser, http://genome.ucsc.edu/, version 1.69); modified according to our study design.

*PTPRE* rs7081735 shows the strongest association to allergic asthma (p = 0.000463) and is the only SNP shown to be significant after multiple testing correction (corrected p = 0.0478). We thus sequenced strategic genomic regions around the rs7081735, including coding regions in order to identify causal mutation. Figure 2 shows the *PTPRE* sequenced regions, the rs7081735 localization and the nine identified variants (diamonds), including four novel ones (c.86172A>G, c.140901G>A, c.140903G>A and c.141102C>T) (Table 4). It is worth to note that any of these identified polymorphisms may affect either *PTPRE* isoforms. A family-based association analysis has been performed between the three studied phenotypes and variants presenting a MAF over 0.05 that were not included in the first genotyping panel, which was the case for four of the nine identified variants (rs7911506, c.86172A>G, rs7895103 and c.140901G>A). Only rs7895103 showed a modest association with asthma, at a level that does not provide additional insights (p = 0.013, compared to p = 0.000463 for rs7081735).

**Table 4:** *PTPRE* sequenced regions and characteristics of identified SNPs.

Sequenced regions [bp] *	Identified SNP †	Base change	MAF (SLSJ) ‡	MAF (CEU) §	SNP location *
1 [75542–75977]	*No SNPs found*				
2 [85761–86205]	**rs7911506**	C>T	0.49	0.296	Intron 2
	**c.86172 A>G**	A>G	0.31	NA	Intron 2
3 [88011–88457]	rs4369314	G>A	0.17	0.192	Intron 2
4 [92004–92507]	rs7081735	A>G	0.47	0.342	Intron 2
	**rs7895103**	T>C	0.05	0.217	Intron 2
5 [134156–134591]	*No SNPs found*				
6 [140684–141152]	rs4262642	C>A	0.19	0.25	Intron 3
	**c.140901 G>A**	G>A	0.40	NA	Intron 3
	c.140903 G>A	G>A	0.03	NA	Intron 3
	c.141102 C>T	C>T	0.03	NA	Intron 4

SNPs marked in bold have been genotyped and analyzed.

Abbreviations used: bp = base pairs, SNP = single nucleotide polymorphism, MAF = Minor allele frequency, SLSJ = Saguenay-Lac-St-Jean, CEU = CEPH Utah residents with ancestry from northern and western Europe, NA = Not available.

* Base pair numbering according to NCBI (http://www.ncbi.nlm.nih.gov/) genome assembly (Build 36.2).

† If known from NCBI SNP and Ensembl (http://www.ensembl.org/index.html) databases, reported by the NCBI rs#.

‡ Calculated on 39 individuals originating from the SLSJ population.

§ Obtained from the HapMap international project database (http://www.hapmap.org/).

### Replication Study

Considering that the *PTPRE* rs7081735 association to allergic asthma is the strongest in the SLSJ sample, we evaluated it in an independent familial cohort, the Childhood Asthma Management Program (CAMP) [34]. The genotyping completion rate was 96% and no discordance was observed upon repeat genotyping of two random plates. The minor allele frequency was 0.33 and was in Hardy-Weinberg equilibrium (p = 0.90). Family-based association showed no significant association for asthma or atopy phenotypes (data not shown). However, to ensure that the selection of the CAMP study for the replication is appropriate and to demonstrate that the association found could result from a childhood subset of asthma instead of an adulthood one, we stratified the SLSJ association analyses considering only the probands that reported an asthma age of onset below 12 years old. Thus, the positive association found for *PTPRE* rs7081735 and allergic asthma (p = 0.000463, Table 3) remains significant when considering only those probands (Family number = 74; Z for the minor allele = −3.166; p = 0.001546). Even with the loss of statistical power due to the stratified analysis, this comparison allowed to assume that the association found for *PTPRE* is probably more related to a childhood asthma, comforting the choice of the CAMP cohort as a replication study.

## Discussion

This study proposes a novel approach for the selection of candidate genes for asthma association studies using the computational G2D tool to find genetic determinants for this disease. Based on a genome-wide scan performed in an asthmatic familial sample from the SLSJ founder population, we selected the two best-linked regions (6q26 and 10q26.3) and applied the G2D data mining approach [17], [18] to identify ten candidate genes for an association in the SLSJ sample. Among these, five (*LPA*, *ADAM8*, *DOCK1*, *GPR123* and *PTPRE*) presented modest associations with asthma, atopy or allergic asthma. After corrections for multiple testing, only the *PTPRE* rs7081735 association to allergic asthma remained significant. These findings demonstrate that the G2D tool can be useful in the selection of candidate genes for asthma genetic studies.

Because the *PTPRE* association to allergic asthma remained significant after correction for multiple testing, we sequenced strategic regions around the rs7081735, aiming to find the causal mutation. Sequencing allowed the identification of five known and four novel variants. Testing four SNPs with MAF >0.05 did not identify additional associated SNPs with asthma or atopy related phenotypes in the SLSJ sample. Thus, the rs7081735, located in the 5′ untranslated region, could be the causal variant, or be in linkage disequilibrium with an unknown causal mutation.

*PTPRE* is a member of the protein tyrosine phosphatase (PTPs) family, which includes genes that are important regulators of signal transduction pathways involved in various cellular processes such as control of metabolic pathways, cellular adhesion, cell cycle progression and immune response [41], [42]. *PTPRE* encodes two different isoforms, cytoplasmic and transmembrane [43], based on its different promoters [44]. PTPs receptors participate in transmembrane signaling and cellular adhesion processes, whereas intracellular PTPs take part in signal transduction within the cell [45]. For example in mice, PTPε-deficient macrophages present abnormalities in the regulation of the respiratory burst and the production of cytokines in response to bacterial lipopolysaccharide, suggesting a role of the PTPε isoform in inflammation as well as in host defense [46]. However, *PTPRE* has been shown to be highly expressed in peripheral human monocytes and granulocytes, and antigen-receptor stimulation induces the expression of *PTPRE* in activated lymphocytes [47]. These observations and our finding suggest a protective effect of *PTPRE* in allergic asthma, and lead us to hypothesize that this potential protective role could involve the leukocyte cellular processes in the limitation of lung inflammation following an allergen sensitization. Further work is needed to define the *PTPRE* possible role in asthma pathophysiology.

The *PTPRE* rs7081735 association has been evaluated in the independent CAMP cohort. Results showed no positive association for asthma and atopic phenotypes. Taking into account that the SLSJ and the CAMP studies are both family-based designed, well powered [48], [49] and presenting a childhood onset asthma, the *PTPRE* association lack of replication in the CAMP study can be explained by other reasons: natural variability of asthma history [50], [51], differences of proband mean age between samples (SLSJ mean age of 18, and CAMP mean age of 8, respectively), differences in the genetic background of the two populations [52] (SLSJ individuals descend predominantly from French European founders [11]–[16] whereas CAMP individuals come from a white North-American admixed population), or population specific gene-gene and gene-environment interactions [52]. Based on these observations, we conclude that the *PTPRE* rs7081735 association to allergic asthma is more penetrant in the SLSJ population, possibly resulting from an interaction with others genes and/or environmental factors that are more common in this founder population.

In summary, this study demonstrates that the G2D tool can be useful in the prioritization of candidate genes for a complex disease as it allowed us to find a novel asthma genetic association with the *PTPRE* gene in the SLSJ familial asthma sample. This association represents a potential protective factor for asthma pathogenesis, as it is more likely related to a childhood onset asthma. The present genetic study is an example of how the combination of different methodological approaches can be relevant to target asthma genetic determinants and to motivate further genetic and functional investigations.

## Supporting Information

Text S1 (0.03 MB DOC)

Table S1 Genome-wide scan Saguenay-Lac-St-Jean subjects clinical characteristics and studied phenotypes
(0.06 MB DOC)

Table S2 Characteristics of the 91 selected SNPs
(0.25 MB DOC)

Table S3 Taqman PCR conditions
(0.06 MB DOC)

Table S4 Oligonucleotides used for PTPRE sequencing
(0.05 MB DOC)

Table S5 Genes selected by G2D « PHENOTYPE » analysis
(0.07 MB DOC)

Table S6 Genes known or suspected to be associated with asthma that were considered for the G2D «KNOWN GENES» analysis
(0.07 MB DOC)

Table S7 Genes selected by G2D « KNOWN GENES » analysis
(0.06 MB DOC)
