# Supplementary material for: Genes to Diseases (G2D) Computational Method to Identify Asthma Candidate Genes
Source: PLoS One. 2008 Aug 6;3(8):e2907. doi: 10.1371/journal.pone.0002907 (PMC2488373; doi:10.1371/journal.pone.0002907)
Supplement: Text S1 — (0.03 MB DOC) [file pone.0002907.s001.doc]

**Title**

Genes to Diseases (G2D) Computational Method to Identify Asthma Candidate Genes

**Authors**

Karine Tremblay, Mathieu Lemire, Camille Potvin, Alexandre Tremblay, Gary M Hunninghake, Benjamin A Raby, Thomas J Hudson, Carolina Perez-Iratxeta, Miguel A Andrade-Navarro and Catherine Laprise

**Supporting text file**

**Methods**

**Association study: Effective number of independent tests**

Since the SNPs are correlated, as are the three phenotypes studied, accounting for the number of tests using a simple Bonferroni correction is expected to be conservative. An effective number of independent SNPs can be calculated by the method proposed by Li and Ji [S1] implemented in SNPSdP [S2]: this number takes into account the observed r^2^ measure of pairwise linkage disequilibrium between all SNPs. Out of the three phenotypes investigated, an effective number of independent phenotypes was calculated using 10000 simulated replicates of the SLSJ sample of families: genotypes for ten SNPs with minor allele frequencies varying from 5% to 50% were simulated under the null hypothesis of no linkage and no association, for all members of the sample who have DNA. FastSLINK [S3-S5] and SUP [S6] were used to create the replicates. Each replicate of genotypes was then analysed for all three studied phenotypes, as they have been observed in the sample, and the minimum p-value observed over the four phenotypes was stored for each of the SNPs. The effective number of independent phenotypes was then taken to be the ratio between 0.05 and the quantile of order 5% of these minimum p-values.

**References**

S1. Li J, Ji L (2005) Adjusting multiple testing in multilocus analyses using the eigenvalues of a correlation matrix. Heredity 95: 221-227.

S2. Nyholt DR (2004) A simple correction for multiple testing for single-nucleotide polymorphisms in linkage disequilibrium with each other. Am J Hum Genet 74: 765-769.

S3. Ott J (1989) Computer-simulation methods in human linkage analysis. Proc Natl Acad Sci U S A 86: 4175-4178.

S4. Weeks DE, Lathrop GM, Ott J (1993) Multipoint mapping under genetic interference. Hum Hered 43: 86-97.

S5. Cottingham RW, Jr., Idury RM, Schaffer AA (1993) Faster sequential genetic linkage computations. Am J Hum Genet 53: 252-263.

S6. Lemire M (2006) SUP: An extension to SLINK to allow a larger number of marker loci to be simulated in pedigrees conditional on trait values. BMC Genet 7: 40.
