# Supplementary material for: Genes to Diseases (G2D) Computational Method to Identify Asthma Candidate Genes
Source: PLoS One. 2008 Aug 6;3(8):e2907. doi: 10.1371/journal.pone.0002907 (PMC2488373; doi:10.1371/journal.pone.0002907)
Supplement: Table S1 — Genome-wide scan Saguenay-Lac-St-Jean subjects clinical characteristics and studied phenotypes (0.06 MB DOC) [file pone.0002907.s002.doc]

**Table S1**

Genome-wide scan Saguenay–Lac-St-Jean subjects clinical characteristics and studied phenotypes

|  | Families | |
| --- | --- | --- |
|  | Probands (n = 79) | Family Members  (n = 530) |
| Male: Female ratio | 1: 1.2 | 1: 1.2 |
| Mean age in years (range) | 17.8 (4-62) | 40.7 (4-88) |
| Smoking status (n (%)) |  |  |
| Never | 68 (86) | 242 (52) |
| Ex-smoker | 6 (8) | 129 (28) |
| Smoker | 5 (6) | 91 (19) |
| FEV_1_ as % predicted (SD) * | 96.5 (13.1) | 97.6 (20.9) |
| PC_20_ in mg/ml (SD) † | 2.6 (3.8) | 9.4 (5.1) |
| Serum IgE in mg/l (SD) † | 228.0 (3.9) | 122.7 (4.4) |
| Number of Persons with Subphenotypes (%) | | |
| Asthma ‡ | 79 (100) | 189 (40) |
| Atopy § | 68 (86) | 249 (54) |
| AHR ∏ | 64 (81) | 165 (52) |
| IgE ** > 100 μg/l | 58 (73) | 190 (53) |
| IgE > 280 μg/l | 38 (48) | 107 (30) |

* FEV_1_ = Forced expiratory volume in one second.

† Geometric mean; PC_20_ = provocative concentration of methacholine inducing a 20% fall in

FEV_1_.

‡ 466 subjects with clinical status documented for asthma.

§ 462 with skin prick test performed.

∏ AHR = Airway hyperreactivity according to American Thoracic Society criteria (< 8 mg/ml)
 Methacholine test was performed for 316 subjects (age ≥ 12 years).

** IgE level was measured for 356 subjects.
