# Supplementary material for: Genes to Diseases (G2D) Computational Method to Identify Asthma Candidate Genes
Source: PLoS One. 2008 Aug 6;3(8):e2907. doi: 10.1371/journal.pone.0002907 (PMC2488373; doi:10.1371/journal.pone.0002907)
Supplement: Table S2 — Characteristics of the 91 selected SNPs (0.25 MB DOC) [file pone.0002907.s003.doc]

**Table S2**

Characteristics of the 91 selected SNPs

| **Gene *** | **SNP** | **Chromosome position †** | **Genomic location †** | **Techno ‡** | **TagSNP Selection §** | **HWE** | **ME** |
| --- | --- | --- | --- | --- | --- | --- | --- |
| *LPA* NM_005577.2 | rs7770628 | 6:160938164 | Intron 20 | S | Yes | 0.109 | 0 |
|  | rs12175867 | 6:160939128 | Intron 20 | S | Yes | 0.584 | 1 |
|  | **rs9355817** | 6:160953578 | Intron 16 | S | Yes | Failed | NA |
|  | rs12526465 | 6:160988597 | Intron 1 | S | Yes | 0.531 | 0 |
|  | rs1652507 | 6:161002451 | Intron 0 | S | Yes | 1.0 | 0 |
|  | rs1367211 | 6:161002685 | Intron 0 | S | Yes | 0.596 | 0 |
|  | rs1321195 | 6:161004146 | Intron 0 | S | Yes | 0.718 | 0 |
|  | **rs9346833** | 6:161004632 | Intron 0 | S | Yes | Failed | NA |
| *NOX3* NM_015718.1 | rs12665231 | 6:155758289 | Exon14 | S | No | 0.440 | 0 |
|  | rs231951 | 6:155791096 | Intron 9 | S | Yes | 0.755 | 0 |
|  | rs231959 | 6:155797666 | Intron 7 | S | Yes | 0.525 | 0 |
|  | rs3749930 | 6:155802938 | Exon 6 - K171T | S | No | 1.0 | 0 |
|  | rs2235673 | 6:155812124 | Intron 4 | S | Yes | 1.0 | 0 |
|  | rs7754577 | 6:155812942 | Intron 4 | S | Yes | 1.0 | 0 |
|  | rs1016259 | 6:155816650 | Intron 3 | S | Yes | 0.900 | 0 |
|  | rs9480140 | 6:155818406 | Intron 1 | S | Yes | 0.689 | 0 |
| *SNX9* NM_016224.3 | rs2490565 | 6:158246186 | Intron 6 | S | Yes | 0.881 | 0 |
|  | rs3805780 | 6:158249963 | Intron 7 | S | Yes | 0.494 | 0 |
|  | rs9456870 | 6:158263794 | Intron 10 | S | Yes | 0.090 | 0 |
|  | rs9365587 | 6:158264825 | Intron 10 | S | Yes | 0.286 | 0 |
|  | rs11759002 | 6:158265875 | Intron 10 | S | Yes | 0.368 | 0 |
|  | rs11755474 | 6:158275006 | Intron 13 | S | Yes | 0.635 | 0 |
|  | rs6455919 | 6:158279463 | Intron 15 | S | Yes | 0.329 | 0 |
|  | rs912879 | 6:158281887 | Intron 16 | S | Yes | 0.388 | 0 |
| *VIL2* NM_003379.3 | rs3102974 | 6:159112907 | Intron 7 | S | Yes | 0.082 | 0 |
|  | rs744893 | 6:159122228 | Intron 6 | S | Yes | 0.749 | 0 |
|  | **rs11550707** | 6:159126572 | Exon 4 - L75P | S | No | NP | NA |
|  | rs756144 | 6:159127578 | Intron 3 | T | Yes | 0.933 | 1 |
|  | rs3123116 | 6:159130114 | Intron 2 | S | Yes | 0.742 | 1 |
|  | rs9295086 | 6:159134798 | Intron 1 | T | Yes | 0.448 | 0 |
|  | rs7754951 | 6:159151086 | Intron 1 | T | Yes | 0.607 | 0 |
|  | rs1994350 | 6:159158992 | Intron 1 | T | Yes | 0.041 | 1 |
| *VIP* NM_003381.2 | rs1282449 | 6:153104299 | Upstream  5' UTR | T | No | 0.225 | 0 |
|  | rs2756117 | 6:153108601 | Upstream 5' UTR | T | No | 0.219 | 0 |
|  | rs7764067 | 6:153115892 | Intron 2 | T | No | 0.476 | 0 |
|  | rs3823082 | 6:153116015 | Intron 2 | T | No | 0.056 | 0 |
|  | rs688136 | 6:153121754 | Exon 7 | T | No | 0.689 | 1 |
|  | rs671330 | 6:153126927 | Downstream 3' UTR | T | No | 0.053 | 1 |

| *ADAM8* NM_001109.3 | rs2230576 | 10:134926587 | Exon 23 - A846A | S | Yes | 0.234 | 0 |
| --- | --- | --- | --- | --- | --- | --- | --- |
|  | rs11101672 | 10:134931744 | Intron 19 | S | Yes | 0.274 | 0 |
|  | rs2275720 | 10:134932336 | Exon 19 - L657F | S | No | 0.090 | 0 |
|  | rs1131718 | 10:134935744 | Exon 10 - T300T | S | Yes | 0.092 | 0 |
|  | **rs3810960** | 10:134937295 | Exon 5 - L115P | S | No | NP | NA |
|  | rs2275725 | 10:134939025 | Exon 2 - R35W | S | Yes | 0.108 | 0 |
| *DOCK1* NM_001380.3 | rs7092254 | 10:128582862 | Upstream 5' UTR | S | No | 0.035 | 1 |
|  | **rs2720994** | 10:128979119 | Intron 27 | S | Yes | Failed | NA |
|  | rs4751240 | 10:129026399 | Intron 27 | S | Yes | 1.0 | 0 |
|  | rs2296635 | 10:129050448 | Exon 32 | S | Yes | 1.0 | 0 |
|  | rs7899059 | 10:129075564 | Intron 36 | S | Yes | 0.107 | 0 |
|  | rs2229599 | 10:129097419 | Exon 40 | S | Yes | 1.0 | 0 |
|  | **rs2296626** | 10:129106789 | Exon 44 | S | Yes | NP | NA |
|  | rs869801 | 10:129135674 | Exon 50 | S | Yes | 1.0 | 0 |
|  | rs2229603 | 10:129139652 | Exon 51 | S | Yes | 1.0 | 0 |
|  | rs1051039 | 10:129140593 | 3'UTR | S | Yes | 0.155 | 0 |
| *FANK1* NM_145235.3 | **rs4010025** | 10:127575163 | Exon1 | S | No | Failed | NA |
|  | **rs4962499** | 10:127623699 | Intron 1 | S | Yes | Failed | NA |
|  | rs10751537 | 10:127637438 | Intron 1 | S | Yes | 0.836 | 0 |
|  | **rs7894453** | 10:127639645 | Intron 1 | S | Yes | 0.555 | 4 |
|  | rs10901491 | 10:127650767 | Intron 1 | S | Yes | 0.584 | 0 |
|  | rs7918092 | 10:127652925 | Intron 1 | S | Yes | 0.920 | 0 |
|  | rs11244753 | 10:127672950 | Intron 3 | S | Yes | 0.961 | 0 |
|  | rs3736477 | 10:127675081 | Intron 4 | S | Yes | 0.720 | 0 |
|  | rs4962316 | 10:127677666 | Intron 6 | S | Yes | 0.697 | 0 |
|  | **rs17153976** | 10:127687987 | Exon 11 - F343C | S | No | NP | NA |
| *GPR123* NM_001083909.1 | rs7912475 | 10:134739856 | Intron 2 | S | Yes | 0.302 | 0 |
|  | rs4838694 | 10:134748584 | Intron 7 | S | Yes | 0.395 | 0 |
|  | rs11101913 | 10:134757229 | Intron 9 | S | Yes | 1.0 | 0 |
|  | rs11101916 | 10:134762304 | Intron 12 | S | Yes | 0.443 | 0 |
|  | rs11101925 | 10:134763348 | Intron 12 | S | Yes | 0.255 | 0 |
|  | rs9419004 | 10:134764512 | Intron 12 | S | Yes | 0.279 | 0 |
|  | rs9419103 | 10:134765822 | Intron 12 | S | Yes | 0.901 | 0 |
|  | rs736017 | 10:134767287 | Intron 13 | S | Yes | 0.331 | 0 |
|  | rs11101932 | 10:134768842 | Intron 13 | S | Yes | 1.0 | 0 |
|  | rs4838797 | 10:134769155 | Intron 13 | S | Yes | 0.239 | 0 |
|  | rs761777 | 10:134788065 | Intron 13 | S | Yes | 0.729 | 0 |
|  | rs12257731 | 10:134791129 | Intron 14 | S | Yes | 0.520 | 0 |

| *PTPRE* NM_006504.3 | rs1359850 | 10:129675326 | Intron 1 | S | Yes | 0.709 | 0 |
| --- | --- | --- | --- | --- | --- | --- | --- |
|  | rs4350281 | 10:129679923 | Intron 1 | S | No | 0.344 | 0 |
|  | rs11016002 | 10:129680794 | Intron 2 | S | No | 0.331 | 0 |
|  | rs4369314 | 10:129683048 | Intron 2 | S | Yes | 0.385 | 0 |
|  | rs7081735 | 10:129687046 | Intron 2 | S | Yes | 0.195 | 0 |
|  | rs4002572 (or rs4281406) | 10:129690845 | Intron 2 | S | Yes | 0.714 | 0 |
|  | **rs4073016** | 10:129691719 | Intron 2 | S | Yes | Failed | NA |
|  | rs7089539 | 10:129706236 | Intron 2 | S | Yes | 0.304 | 0 |
|  | rs10764735 | 10:129715444 | Intron 2 | S | Yes | 0.211 | 0 |
|  | rs7083801 | 10:129725918 | Intron 2 | S | Yes | 0.574 | 0 |
|  | rs4262642 | 10:129735537 | Intron 3 | S | Yes | 0.122 | 0 |
|  | rs7079639 | 10:129751782 | Intron 10 | S | Yes | 0.126 | 0 |
|  | rs10741142 | 10:129763083 | Intron 17 | S | Yes | 0.401 | 0 |
|  | rs2298197 | 10:129765704 | Intron 18 | S | Yes | 0.105 | 0 |
|  | rs3210509 | 10:129771768 | Exon 21 | S | Yes | 0.401 | 0 |

SNPs marked in bold have not been analyzed for the association study.

Abbreviations used: SNP = Single nucleotide polymorphism; HWE = Hardy-Weinberg Equilibrium (Haploview P-values); ME = Mendelian errors reported in FBAT analyses (number); NA = Not available.

* Gene symbol and the transcript variant used (NM number).

† From NCBI (<http://www.ncbi.nlm.nih.gov/>) SNP database (build 127).

‡ Technology used : S = Sequenom, T = Taqman.

§ Yes = tagSNP selection, No ≠ tagSNP selection.
