# Supplementary material for: Genes to Diseases (G2D) Computational Method to Identify Asthma Candidate Genes
Source: PLoS One. 2008 Aug 6;3(8):e2907. doi: 10.1371/journal.pone.0002907 (PMC2488373; doi:10.1371/journal.pone.0002907)
Supplement: Table S3 — Taqman PCR conditions (0.06 MB DOC) [file pone.0002907.s004.doc]

**Table S3**

Taqman PCR conditions

| **Gene** | **SNPs** | **Assay ID *** |  | **PCR** | |
| --- | --- | --- | --- | --- | --- |
|  |  |  |  | **Mix †** | **Cycling conditions** |
| VIL2 (SLSJ) | rs756144 | AssayID C_8305215_10 |  | 10 ng of genomic DNA, 0.2 units of AmpliTaq GoldTM DNA Polymerase, 0.5 μl of 10x PCR Gold Buffer, concentration of MgCl2 ranging from 1.25 to 4 mM depending on the SNP, 0.25 mM of dNTPs, 0.15 μl of 20X TaqMan SNP Genotyping Assay mix, and water for a final volume of 5 μl. | 95°C 10 min, 45 to 50 cycles of 92°C 15 sec and 58°C 1 min. |
|  | rs9295086 | AssayID C_29990632_10 |  |  |  |
|  | rs7754951 | AssayID C_11551998_10 |  |  |  |
|  | rs1994350 | AssayID C_12033615_10 |  |  |  |
| VIP (SLSJ) | rs1282449 | AssayID C_462198_10 |  |  |  |
|  | rs2756117 | AssayID C_16286919_10 |  |  |  |
|  | rs7764067 | AssayID C_29430252_10 |  |  |  |
|  | rs3823082 | AssayID C_27491244_10 |  |  |  |
|  | rs688136 | AssayID C_3250639_10 |  |  |  |
|  | rs671330 | AssayID C_2144586_10 |  |  |  |
| PTPRE (CAMP) | rs7081735 | AssayID C__29345951_10 |  | 6.25 ng of genomic DNA, 2.5 μl of Applied Biosystems 2x Universal Master Mix, 0.125 μl of 40X TaqMan SNP Genotyping Assay mix, and water for a final volume of 5 μl. | 50°C 2 min, 95°C 10 min, 50 cycles of 95°C 15 sec and 60°C 1 min. |

* Refers to the Taqman kit identification number supplied by the company.

† From Applied Biosystems Inc.
