# Supplementary material for: Genes to Diseases (G2D) Computational Method to Identify Asthma Candidate Genes
Source: PLoS One. 2008 Aug 6;3(8):e2907. doi: 10.1371/journal.pone.0002907 (PMC2488373; doi:10.1371/journal.pone.0002907)
Supplement: Table S4 — Oligonucleotides used for PTPRE sequencing (0.05 MB DOC) [file pone.0002907.s005.doc]

**Table S4**

Oligonucleotides used for *PTPRE* sequencing

| Oligo name * | Covered region | Product size | Sequence (5’ – 3’) † |
| --- | --- | --- | --- |
| PTPRE_Exon2_L | Exon 2 | 436 | CACAGCAGAGGCAGCTGAC |
| PTPRE_Exon2_R |  |  | CCTGCTCAAGCAAGGTAAGG |
| PTPRE_rs11016002_L | Around rs11016002 | 445 | GGGGAAAACAACATGACCAG |
| PTPRE_rs11016002_R |  |  | GTGTCTGGCTTTCTGATGCA |
| PTPRE_rs4369314_L | Around rs4369314 | 447 | AGAACGCAGGCCCCATAG |
| PTPRE_rs4369314_R |  |  | TTGGATTGGTGGGTACATCC |
| PTPRE_rs7081735_L | Around rs7081735 | 504 | GGCTTTCCATTTCTGTCTGG |
| PTPRE_rs7081735_R |  |  | CCCCTTCTACAGCACTTCCTAA |
| PTPRE_Exon3_R | Exon 3 | 436 | TGGGTTCCAGTCCCTGATC |
| PTPRE_Exon3_L |  |  | AGCCCCTTTATCACCCTCTG |
| PTPRE_Exon4_L | Exon 4 | 468 | GGGAACTCACCCCTGTCAT |
| PTPRE_Exon4_R |  |  | CCTGGCCTCTGTGTGTCTC |

* Annealing temperatures for each oligo pair is 64°C.

† PCR were done in 25µL volume containing of 0.6 unit of Taq DNA polymerase (New England Biolabs), 50µM of each dNTPs, 20 mM Tris-HCl, 10 mM (NH_4_)_2_SO_4_, 10 mM KCl, 2 mM MgSO_4_, 0.1 % Triton, pH 8.8, 0.4µM of each primer and 10 *n*g of human genomic DNA amplified with the following conditions: 95°C 15 min followed by 35 cycles of 94°C 30 sec, 59-66°C 40 sec and 72°C 1 min, then 72°C 5 min.
