# Supplementary material for: Genes to Diseases (G2D) Computational Method to Identify Asthma Candidate Genes
Source: PLoS One. 2008 Aug 6;3(8):e2907. doi: 10.1371/journal.pone.0002907 (PMC2488373; doi:10.1371/journal.pone.0002907)
Supplement: Table S6 — Genes known or suspected to be associated with asthma that were considered for the G2D «KNOWN GENES» analysis (0.07 MB DOC) [file pone.0002907.s007.doc]

**Table S6**

Genes known or suspected to be associated with asthma that were considered for the G2D «KNOWN GENES» analysis

| Gene ID * | Symbol † | Description | GO annotation ‡ |
| --- | --- | --- | --- |
| 387129 | GPRA | G protein-coupled receptor 154 | No |
| 404744 | AAA1 | Asthma associated protein (MMistery protein) | No |
| 3596 | IL13 | Interleukin 13 | Yes |
| 154 | ADRB2 | adrenergic, beta-2-, receptor | Yes |
| 3593 | IL12B | interleukin 12B (natural killer cell stimulatory factor 2, cytotoxic lymphocyte maturation factor 2, p40) | Yes |
| 7356 | SCGB1A1 | secretoglobin, family 1A, member 1 | Yes |
| 7124 | TNF | tumor necrosis factor (TNF superfamily, member 2) | Yes |
| 51131 | PHF11 | PHD finger protein 11 | No |
| 117156 | SCGB3A2 | secretoglobin, family 3A, member 2 | Yes |
| 2206 | MS4A2 | membrane-spanning 4-domains, subfamily A, member 2 | Yes |
| 5729 | PTGDR | prostaglandin D2 receptor (DP) | Yes |
| 4056 | LTC4S | leukotriene C4 synthase | Yes |
| 3567 | IL5 | interleukin 5 (colony-stimulating factor, eosinophil) | Yes |
| 2944 | GSTM1 | glutathione S-transferase M1 | Yes |
| 80332 | ADAM33 | ADAM metallopeptidase domain 33 | Yes |
| 3565 | IL4 | interleukin 4 | Yes |
| 3566 | IL4R | interleukin 4 receptor | Yes |
| 57628 | DPP10 | dipeptidyl-peptidase 10 | Yes |
| 727 | C5 | complement component | Yes |

* Entrez Gene identifier in the NCBI database (<http://www.ncbi.nlm.nih.gov/>).

† Gene symbol.

‡ Genes not annotated with GO terms could not be used for this analysis.
